# Supplementary figures and images for: Therapeutic potential of salidroside in type I diabetic erectile dysfunction: Attenuation of oxidative stress and apoptosis via the Nrf2/HO-1 pathway
Source: PLoS One. 2024 Jul 11;19(7):e0306926. doi: 10.1371/journal.pone.0306926 (PMC11238988; doi:10.1371/journal.pone.0306926)

Sham DM DM+L DM+H

Nrf2

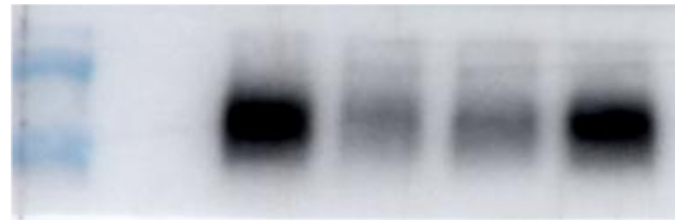

110kDa

HO-1

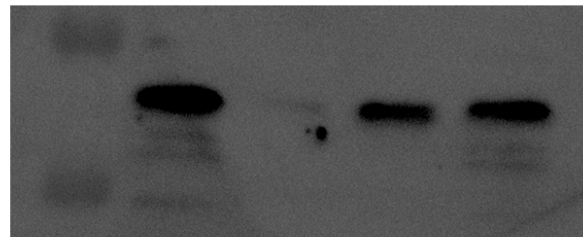

32kDa

Bcl-2

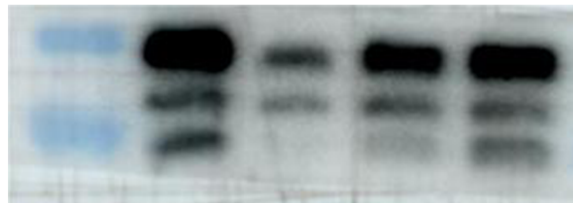

26kDa

Bax

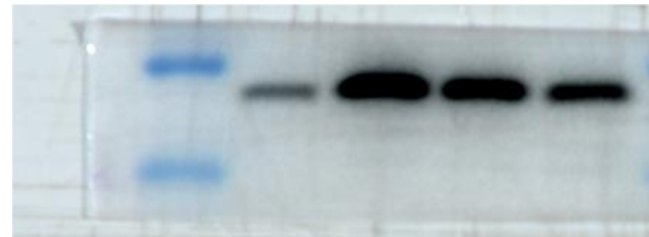

21kDa

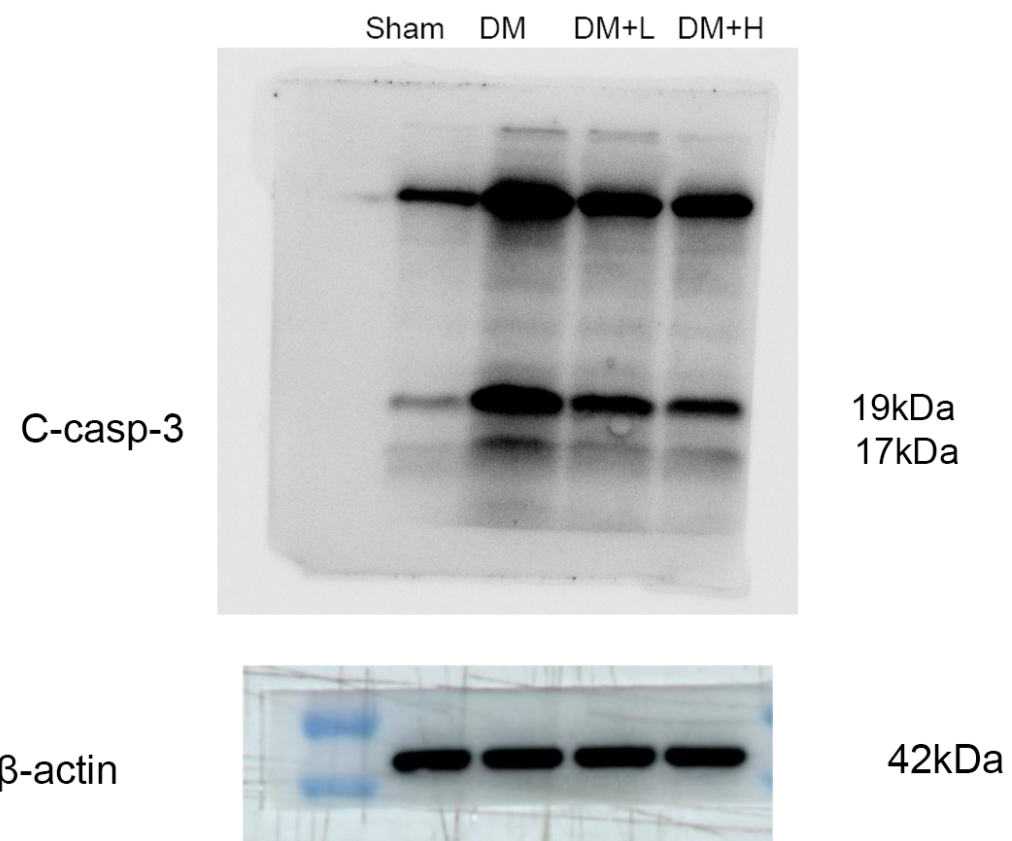

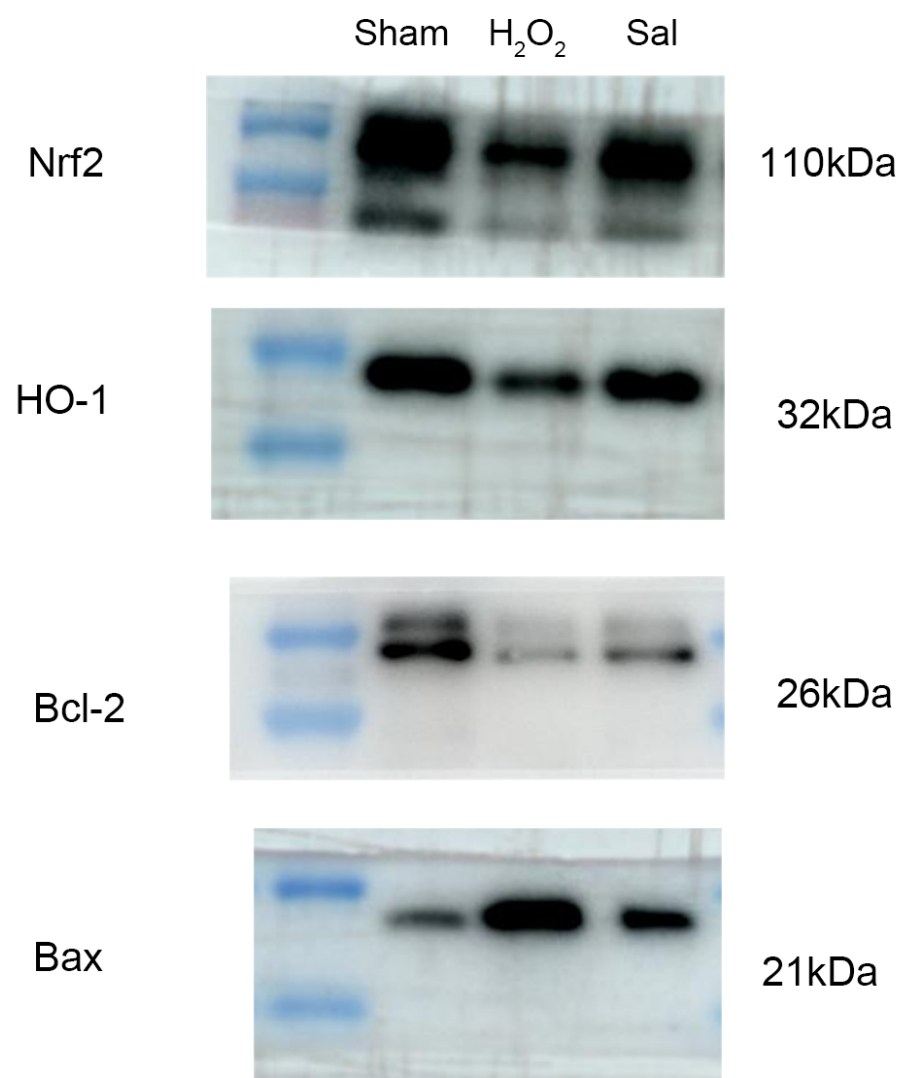

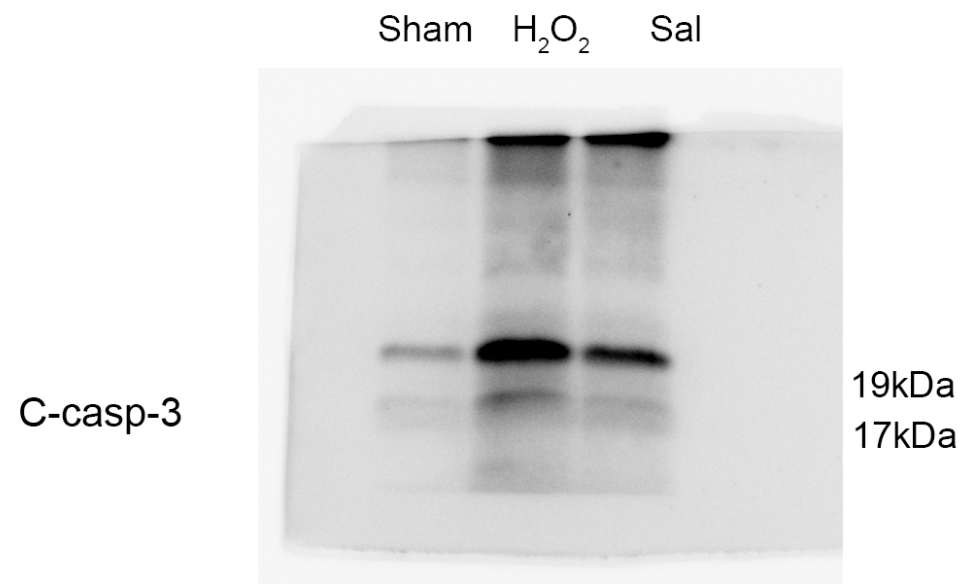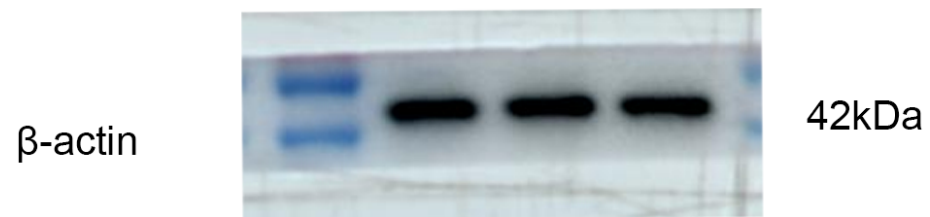

Supplement: S1 Raw images — (PDF) [file pone.0306926.s009.pdf]
