# Supplementary material for: Therapeutic potential of salidroside in type I diabetic erectile dysfunction: Attenuation of oxidative stress and apoptosis via the Nrf2/HO-1 pathway
Source: PLoS One. 2024 Jul 11;19(7):e0306926. doi: 10.1371/journal.pone.0306926 (PMC11238988; doi:10.1371/journal.pone.0306926)
Supplement: Attachment — Submitted filename: Recommendation.docx [file pone.0306926.s010.docx]

**Manuscript Reviews for PLOS ONE**

**Manuscript Title: Therapeutic Potential of Salidroside** 1 **in Diabetic Erectile Dysfunction:** **Attenuation of Oxidative Stress and Apoptosis via the Nrf2/HO-1** Pathway (PONE-D-24-00064)

**Authors:** Zhenghao Li et al.

**Comments to authors:**

I am very much delighted to review the manuscript (PONE-D-24-00064) and found that the authors have put their valuable efforts and knowledge to do an innovative research and have prepared the manuscript nicely.

The manuscript has been written following clear English language and grammar. The Introduction chapter contains relevant background literature and well-referenced information. All the figures, table and data have been provided which are statistically measured and presented.

Experimental design and methods are well described. The analytical parameters are adequate for the interpretation and discussion of the results. Research questions are well-defined and explored. Results indicate that salidroside upregulated the Nrf2/HO-1 signalling pathway, leading to the improvement of erectile function in diabetic male rats by alleviating oxidative stress and reducing apoptosis in corpus cavernosum tissue. This is an original research paper so far. Preprint of the same article is available at <https://doi.org/10.21203/rs.3.rs-3446173/v1>.

The conclusion summarizes findings, and discusses the implications. Over all, the manuscript can be accepted for publication.
