# Supplementary material for: Therapeutic potential of salidroside in type I diabetic erectile dysfunction: Attenuation of oxidative stress and apoptosis via the Nrf2/HO-1 pathway
Source: PLoS One. 2024 Jul 11;19(7):e0306926. doi: 10.1371/journal.pone.0306926 (PMC11238988; doi:10.1371/journal.pone.0306926)
Supplement: Attachment — Submitted filename: Response to Reviewers.docx [file pone.0306926.s011.docx]

Dear Reviewers,

We would like to express our sincere gratitude for your insightful and constructive feedback on our manuscript titled "[Manuscript Title]" (PONE-D-24-00064). Your comments have been invaluable in enhancing the quality and clarity of our work, and we truly appreciate the time and effort you have dedicated to reviewing our manuscript.

To Reviewer #1:

We are delighted that you found our research innovative and well-prepared. Your positive assessment of the clarity of language, adequacy of experimental design and methods, and the relevance of our findings to the field is truly encouraging. We are grateful for your acknowledgment of the originality of our research and your recognition of the significance of our findings. We have duly noted your comments regarding the availability of the preprint version of our manuscript and will ensure that any necessary updates or revisions are reflected in the final published version.

To Reviewer Dr. Md. Masudur Rahman:

We appreciate your thoughtful comments and suggestions for further improvement of our manuscript. We agree with your suggestion to consider rephrasing the title to specify the type of diabetic erectile dysfunction (DMED) studied in our research. In addition, we believe that the results of Western blot demonstrate a change regarding the level of the Nrf2 pathway from the protein expression level, which is more intuitive than qRT-PCR analysis of the gene. Regarding the concluding remarks, we acknowledge that the statement about activation of the Nrf2 pathway should be supported by further evidence and will address this issue accordingly in the revised manuscript.

In response to your feedback, we have already revising the manuscript to address the specific points raised by both reviewers. We are confident that these revisions will enhance the clarity, specificity, and overall quality of the manuscript.

Once again, we extend our sincere appreciation for your valuable feedback and constructive criticism. We are committed to addressing all concerns and ensuring that our manuscript meets the standards of PLOS ONE. We look forward to submitting the revised version for your consideration.

Thank you for your continued support and guidance throughout this review process.

Best regards,

Zhenghao Li
